# Curcumin and Capsaicin-Loaded Ag-Modified Mesoporous Silica Carriers: A New Alternative in Skin Treatment

**DOI:** 10.3390/nano12173075

**Published:** 2022-09-05

**Authors:** Ivalina Trendafilova, Ralitsa Chimshirova, Denitsa Momekova, Hristo Petkov, Neli Koseva, Penka Petrova, Margarita Popova

**Affiliations:** 1 Institute of Organic Chemistry with Centre of Phytochemistry, Bulgarian Academy of Sciences, 1113 Sofia, Bulgaria; 2 Faculty of Pharmacy, Medical University of Sofia, 1000 Sofia, Bulgaria; 3 Institute of Polymers, Bulgarian Academy of Sciences, 1113 Sofia, Bulgaria; 4 Institute of Microbiology, Bulgarian Academy of Sciences, 1113 Sofia, Bulgaria

**Keywords:** silver-modified silica, mesoporous carrier, curcumin, capsaicin, drug delivery, antioxidant activity, cytotoxicity

## Abstract

Biologically active substances of natural origin offer a promising alternative in skin disease treatment in comparison to synthetic medications. The limiting factors for the efficient application of natural compounds, such as low water solubility and low bioavailability, can be easily overcome by the development of suitable delivery systems. In this study, the exchange with the template procedure was used for the preparation ofa spherical silver-modified mesoporous silica nanocarrier. The initial and drug-loaded formulations are fully characterized by different physico-chemical methods. The incipient wetness impregnation method used to load health-promoting agents, curcumin, and capsaicin in Ag-modified carriers separately or in combinationresulted in high loading efficiency (up to 33 wt.%). The interaction between drugs and carriers was studied by ATR-FTIR spectroscopy. The release experiments of both active substances from the developed formulations were studied in buffers with pH 5.5, and showed improved solubility. Radical scavenging activity and ferric-reducing antioxidant power assays were successfully used for the evaluation of the antiradical and antioxidant capacity of the curcumin or/and capsaicin loaded on mesoporous carriers. Formulations containing a mixture of curcumin and capsaicin were characterized bypotentiation of their antiproliferative effect against maligning cells, and it was confirmed that the system for simultaneous delivery of both drugs has lower IC_50_ values than the free substances.The antibacterial tests showed better activity of the obtained delivery systems in comparison with the pure curcumin and capsaicin. Considering the obtained results, it can be concluded that the obtained delivery systems are promising for potential dermal treatment.

## 1. Introduction

For centuries, humans have relied on the power of natural products to deal with different conditions and diseases. Scientific and technological progress opens doors to significantly more efficient utilization and application of products of natural origin in pharmaceutics, cosmetics, and dietary supplements. As the biggest organ in the human body, the skin is the body’s “shield”, protecting it from the aggressive influence of the environment; this makes it extremely vulnerable. It can be affected not only by external factors, butalso by internal body disruptions. There are different routes of medication administration for the treatment of skin diseases. The commonly used skin-directed therapies are applied externally on the skin (topical medications (steroids), photo, and radiation therapy). They are mostly active on the surface of the skin, without much absorption into the bloodstream. Synthetic topical medicines’ drawbacks are commonly associated with several side effects, such as thinning of the skin, redness, irritation, and allergy (dermatitis). In addition, conventional drugs are distributed non-specifically in the body, where they affect not only the target cells (for example, cancerous cells) but also healthy cells, resulting in dose-related side effects and inadequate drug concentrations reaching the target [1]. The approval for clinical application of a therapeutic agent requires a proven safety profile of the substances for human consumption, and on this basis, an excellent alternative to synthetic bioactive compounds isfood-derived natural components such as curcumin and capsaicin, which attract the increasing interest of many research groupsworldwide. Curcumin ((1E,6E)-1,7-Bis(4-hydroxy-3-methoxyphenyl)hepta-1,6-diene-3,5-dione) is the main bioactive compound found in turmeric (*Curcuma longa*) and has been widely used as a traditional medicine for centuries [2,3,4]. Due to its low toxicity and pharmacological activity, its application in the treatment of various diseases, such as cancer [5,6,7,8], inflammatory diseases [9,10], and diabetes [11,12], has been the focus of extensive studies. Capsaicin (trans-8-methyl-N-vanillyl-6-nonenamide) is a natural substance and the major active ingredient of the chili pepper (*Capsicum annuum*) [13]. It has been used medicinally for centuries for its ability to reduce cholesterol, blood lipid, and sugar content and its antioxidative, anti-inflammatory, antiobesity, and analgesic properties [14,15,16,17,18]. Studies proved capsaicin’s potential as an antitumor compound in a wide variety of cancer types, including breast, lung, prostate, gastric, liver, and bladder cancer [19,20,21,22,23,24]. It isused to treat neuropathic pain without the number of side effects seen in the use of oral medications [25]. The pungent alkaloid capsaicin is able to improve the permeability of nutrients in the gastrointestinal system [26] and acts as a co-adjuvant for potentiating the permeability of curcumin [27]. In combination, both biologically active compounds (BAC) demonstrate synergisticanti-inflammatory properties superior tohalf of the individual concentration of curcumin and capsaicin [28]. The mechanism of action responsible for the beneficial biological activity of these two well-known natural compounds hasbeen extensively studied and reported [29,30,31,32,33,34]; however, the use of the most natural compounds, including curcumin and capsaicin, in clinical practice is greatly limited due to their low water solubility, limited tissue distribution, low bioavailability, and short half-life because of extensive metabolism [35,36]. In our previous work, we showed that these problems couldbe easily overcome with the incorporation of biologically active compounds in different mesoporous silica carriers. In the recent study, we chose to modify the silica carriers with silver, not only because of its antibacterial (antimicrobial) properties and possible beneficial effects on damaged skin [37,38,39,40,41], but also because of its ability to form complexes with curcumin [42,43,44] and capsaicin [45,46], which is a premise for more efficient loading of these compounds in the pores of silica.

In the present work, silver-modified mesoporous silica nanocarriers, prepared by the template ion-exchange method, were loaded with curcumin, capsaicin, or a mixture of both. For a more detailed study ofthe influence of the modifying agent on the properties of the delivery system, a sample of pure silica loaded with a mixture of the two bioactive compounds was prepared and analyzed. In vitro release profiles of loaded biologically active compounds from non- and Ag-modified silica particles were studied withrespect totheir possible application as dermal formulations. The antioxidant activity of pure and loaded curcumin and capsaicin was investigated by two different approaches—the ferric-reducing antioxidant power (FRAP) assay and DPPH free radical scavenging activity assay. The cytotoxic potential of the obtained delivery systems was evaluated against human cell type HUT-78 cells as a model of cutaneous T-cell lymphoma (CTCL)in vitro.

## 2. Materials and Methods

### 2.1. Chemicals

Cetyltrimethyl ammonium bromide (CTAB), Pluronic P-123, 2,2-Diphenyl-1-picrylhydrazyl (DPPH), tetraethyl orthosilicate (TEOS), curcumin (Curc), and capsaicin (Caps) were purchased by Sigma-Aldrich, Schnelldorf, Germany; Iron (II) sulfate heptahydrate (analytical grade) from Valerus, Sofia, Bulgaria; Iron (III) chloride (98%) and 2,4,6-tri(2-pyridyl)-1,3,5-triazine (99%) from Acros Organics, Geel, Belgium;sodium acetate trihydrate from Chim-spectar, Sofia, Bulgaria; acetic acid, (≥99.8%) from Honeywell, Offenbach am Main, Germany, and used without further purification.

### 2.2. Synthesis of Spherical Mesoporous Silica

The mesoporous silica with spherical morphology of the particles was obtained according to procedure based on the application of two structure-directing agents (templates)—CTAB and Pluronic 123 [47]. First, two separated solutions of each template were prepared as follows: (1) 3 g Pluronic 123 were dissolved in 60 mL 1.5 M HCl, and (2) 0.6 g CTAB was fully dissolved in 25 mL distilled H_2_O. Second, solutions (1) and (2) were then mixed, and 20 mL ethanol and 10 mL TEOS were additionally added. The obtained mixture was stirred for 45 min at 35 °C. After that, the mixture was transferred in Teflon-lined autoclaves and aged at 80 °C for 48 h. The obtained white precipitate was separated by filtration, washed with distilled H_2_O, and dried at ambient temperature. To remove the template and to obtain free-pore particles, the material was calcinated at 550 °C for 6 h, with a heat rate of 1 °C/min. The product was assigned as sMS.

### 2.3. Modification of Spherical Mesoporous Silica with Silver by Template Ionexchange

The synthesized template-containing spherical mesoporous silica was impregnated with 4 wt.% silver (withrespect to the mass of the template-free silica) using the template ion-exchange approach [48]. For this purpose, 0.318 g AgNO_3_ wasdissolved in 200 mL distilled H_2_O, and then to the solution wasadded 8.7 g spherical mesoporous silica (corresponding to 5 g template-free spherical mesoporous silica). The mixture was stirred at 80 °C for 20 h. The obtained product was collected by filtration, washed with distilled H_2_O, and dried at ambient temperature. For full removal of the organic templates, silver-containing silica was further calcined at 550 °C for 6 h, with a heat rate of 1 °C/min. Thus, obtained material was denoted as Ag-spherical mesoporous silica (AgsMS).

### 2.4. Loading of Biologically Active Compounds (BAC)

Pure and Ag-containing spherical mesoporous silica (sMS, AgsMS) were loaded by the incipient wetness impregnation method at a mass ratio of 1:2 for the biologically active compounds (curcumin and capsaicin) to the carrier. This ratio was proved optimal in our previous studies [49]. After solubility tests of the applied substances, absolute ethanol was chosen as the most suitable solvent, which, in addition to providing complete dissolution, was also preferred due to its low toxicity. In addition, the low boiling point of absolute ethanol allows fast evaporation, even at room temperature, which optimizes the production time of the delivery systems. The powdered capsaicin or curcumin was stirred till complete dissolution in a minimal quantity of EtOH, and then the dried Ag-containing carriers were added. The mixture was magnetically stirred up to the total evaporation of the solvent. The obtained samples were donated AgsMS-Curc andAgsMS-Caps. In order to study possible synergism [28,50], the following systems containing a mixture of both BAC were prepared additionally: AgsMS-CurcCaps and sMS-CurcCaps. As for the systems containing both compounds, BAC:carrier ratio stayedat 0.5, and Curc:Caps mass ratio was 1:1.

### 2.5. Materials Characterization

#### 2.5.1. X-ray Powder Diffraction (XRD)

The structure and crystallinity of the obtained pure and Ag-modified silica, pure curcumin, and capsaicin, as well as all drug delivery systems, were characterized by X-ray Powder Diffraction using Bruker D8 Advance diffractometerequipped with Cu Kα radiation and LynxEye detector (Bruker, Karlsruhe, Germany). The XRD patterns were collected within the range of 1–80° with a constant step of 0.02° and a counting time of 1 s/step.

#### 2.5.2. N_2_-Physisorption

N_2_-physisorption was used for the determination of the surface area, pore volume, and pore size distribution of all carriers and obtained delivery systems. The adsorption-desorption isotherms were collected at −196 °C using AUTOSORB iQ-C-MP-AG-AG (Quantachrome Instruments, Boynton Beach, FL, USA). Beforehand, pure silica and Ag-containing materials were outgassed under vacuum for 2 h at 200 °C, while drug-loaded samples were pretreated at 80 °C for 6 h [51,52]. The specific surface areas were calculated by applying the Brunauer–Emmett–Teller (BET) theory [53]. The total pore volume of all samples was estimated on the basis of the amount of nitrogen adsorbed at a relative pressure of 0.99 [54]. The average pore sizes were calculated by the Barrett–Joyner–Halenda (BJH) method from adsorption isotherms [55].

#### 2.5.3. Transmission Electron Microscopy (TEM) and Scanning Electron Microscope (SEM)

The morphology and the size of the pure silica and Ag-modified particles were examined by transmission electron microscopy and scanning electron microscope. TEM images were taken on MORGAGNI 268D 100 kV; W filament; point-resolution = 0.5 nm apparatus (FEI, Hillsboro, OR, USA). Powdered samples were dispersed in a small amount of ethanol and a drop of this dispersion was placed on a copper grid covered by carbon supporting film and dried at ambient temperature. The SEM analysis wascarried out on Philips 515 instrument (FEI, Hillsboro, OR, USA) working at 20 kV accelerating voltage. The samples were covered with 10 nm layer of gold before investigation.

#### 2.5.4. Thermogravimetric Analysis (TGA)

The content of bioactive organic compounds loaded in porous silica carriers was determined by a thermogravimetric analysis performed on STA449F5 Jupiter instrument (NETZSCH Gerätebau GmbH, Selb, Germany)at a temperature from 25 to 600 °C with a heating rate of 5 °C/min in airflow. The presented results were corrected as the amount of water observed in the pure silica sample was subtracted.

#### 2.5.5. Attenuated Total Reflection Fourier Transform Infrared Spectroscopy (ATR-FTIR)

The interactions of organic molecules (curcumin, capsaicin) with the surface groups of the parent and Ag-containing silica particles were studied with the attenuated total reflection infrared method. ATR-FTIR spectra were recorded by means of IRAffinity-1 Fourier Transform Infrared spectrophotometer (Shimadzu Europa GmbH, Duisburg, Germany) with MIRacle Attenuated Total Reflectance Attachment. The instrument is equipped with a temperature-controlled, high-sensitivity DLATGS detector and ATR attachment with a KRS-5 prism, both purchased from Shimadzu Europa GmbH (Duisburg, Germany). For each sample, 50 scans with 4 cm^−1^ resolution were applied, and the spectral data wereprocessed with an IR solution software.

### 2.6. In Vitro Release Study

The obtained systems were designed for potential topical application of the loaded components (Curc, Caps, Ag). In this regard, the release profiles of the BAC were studied in vitroin citrate buffer at pH = 5.5 at 35 °C, which are optimal conditions for topical drug forms testing according to the European Pharmacopoeia. The drug delivery systems (5 mg, equivalent to ~1.65 mg loaded drug) or pure drug (2 mg) were incubated in 100 mL release media (pH = 5.5) at 37 °C under continuous stirring. At time intervals 5 to 360 min, aliquots of the release medium were analyzed with Shimadzu UV-1280 spectrophotometer (Shimadzu Europa GmbH, Duisburg, Germany) at a wavelength of 425 nm for Curc and 280 nm for Caps, respectively [56,57]. After the analysis, the sample was returned to the flask to avoid the effects of reducing the acceptor phase volume. The concentration of the released BAC was calculated according to the standard curve at pH = 5.5 (R^2^ > 0.9993).

### 2.7. Antioxidant Activity Assays

#### 2.7.1. Ferric-Reducing Antioxidant Power (FRAP) Assay

**Scheme 1:**
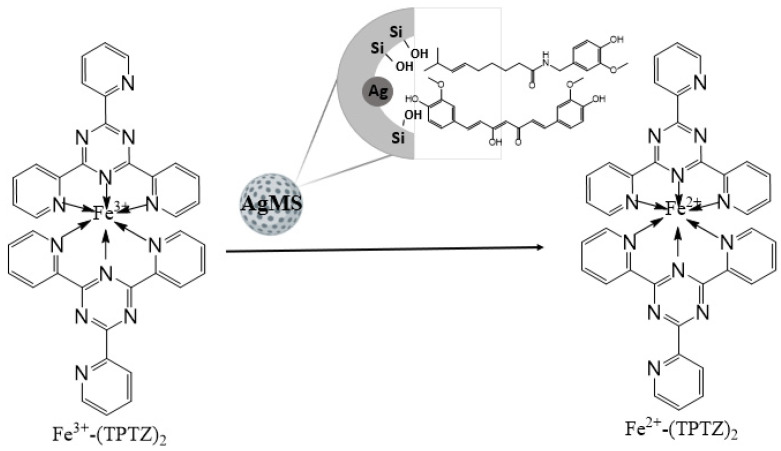
Reaction of the Fe^3+^-(TPTZ)_2_complex with an antioxidant generates the reduced form Fe^2+^-(TPTZ)_2_.

This sensitive and simple technique is commonly used to evaluate the antioxidant capacity of biological fluids, plant extracts, and pure compounds. In its essence, ferric iron complexed to 2,4,6-tri(2-pyridyl)-1,3,5-triazine (TPTZ) acts both as an oxidant and a chromophore. The reaction of the Fe^3+^-(TPTZ)_2_ complex with an antioxidant generates the reduced form Fe^2+^-(TPTZ)_2_ that absorbs light at around 600 nm [58] (Scheme 1).

The assay was performed according to Benzie and Strain procedure [59] with slight modifications. The fresh FRAP reagent was prepared as follows: mixing 0.3 M acetate buffer, 10 mM TPTZ in 40 mM HCl, and 20 mM FeCl_3_·6H_2_O in distilled H_2_O, in a ratio of 10:1:1. The ferric-reducing antioxidant power was evaluated by the mixing of 3 mL FRAP reagent with 100 μL of the investigated sample (dissolved and diluted with ethanol to a concentration of 0.1 mg/mL). The reaction mixture was left for 30 min at room temperature in darkness. After that, the absorbance was measured at 593 nm, and the FRAP value was calculated from a calibration curve of FeSO_4_·7H_2_O standard solutions and expressed as µmol Fe^2+^/L.

#### 2.7.2. DPPH Free Radical Scavenging Activity

**Scheme 2:**
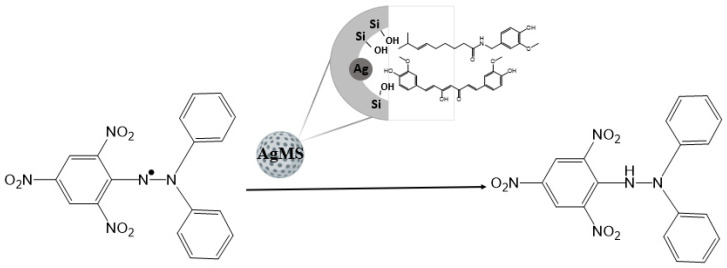
DPPH free radical scavenging activity.

The radical scavenging activity (RSA) of the pure compounds and the loaded delivery systems against free DPPH radicals was determined according to a previously described procedure [60] (Scheme 2). Fresh methanolic DPPH solution (2 mL, 0.1 mM) was mixed with 100 μL ethanolic solution (0.1 mg/mL) of each tested sample. The decrease in the absorption was measured after 30 min storage in a dark place at 517 nm using a Shimadzu UV-1280 spectrophotometer.

Pure curcumin and capsaicin, as well as a mixture of both (1:1, *v*/*v*), were used as positive controls (0.1 mg/mL). Blank solutions were used to correct the absorbance values for radical decay. The values of RSA of the tested samples were given as a percentage with respect to a control value and calculated by the following equation:

RSA (%) = [(A_0_ − A_s_)/A_0_] × 100

where A_0_ is the absorbance of the control sample, and A_s_ is the absorbance of the tested sample.

### 2.8. Evaluation of Cytotoxic Potential

#### 2.8.1. Cell Lines and Culture Conditions

The cytotoxic potential of free active drugs and their mesoporous silica-based formulations was evaluated on HUT-78 cells (cutaneous T-cell lymphoma, CTCL-Cesary syndrome). The cell line was obtained from the German Collection of Microorganisms and Cell Cultures (DSMZ GmbH, Braunschweig, Germany). The cells were cultured in RPMI-1640 supplemented with 10% FBS at 37 °C and humidified atmosphere (5% CO_2_) in an incubator ‘BB 16-FunctionLine’ Heraeus (Kendro, Hanau, Germany). 

#### 2.8.2. MTT-Assay

The growth-inhibitory effects of free curcumin and capsaicin and their loaded silica formulations were evaluated using an MTT-dye reduction assay against HUT-78. Shortly, the cells were seeded in 96-well flat-bottomed plates (100 µL/well) at a density of 1 × 105/mL and kept for 24 h at 37 °C. Afterward, the cells were exposed for 72 h to increasing concentrations (0.02 to 0.1 mg/mL) of the tested formulations or free drugs (as ethanol solutions). Cells from at least sixwells were treated for each individual concentration. After the treatment period, 100 μL of MTT solution (10 g·L^−1^ in PBS) wasadded to each well and the plates were further incubated for 4 h at 37 °C. The formed MTT-formazan crystals were dissolved using 5% formic acid-acidified 2-propanol. The MTT-formazan absorption was measured at 580 nm using a LabeximLMR-1 microplate reader (Labexim International, Lengau, Austria). Cell survival fractions were presented as a percentage of the untreated control and the IC_50_ values were derived from the corresponding dose-response curves.

### 2.9. In Vitro Antibacterial Activity

The antibacterial activity of new nanomaterials was measured by the agar well diffusion method. As test microorganisms were used *Escherichia coli* ATCC 25922, *Pseudomonas aeruginosa* ATCC 9027, and *Staphylococcus aureus* ATCC 6538. The test microorganisms’ cultures were grown in Luria-Bertani (LB) medium for 24 h at 37 °C and diluted with sterile saline (0.9% NaCl) to 106 colony-forming units per mL (CFU/mL). Then, 50 μL of the cell suspensions were mixed with 20 mL LB-agar (45 °C) in Petri dishes. An amount of 100 μL of sterile water-diluted substances with appropriate concentrations wasplaced in agar wells (10 mm in diameter). The agar plates were incubated at 12 °C for 4 h to allow substances diffusion and then for 24 h at 37 °C.

The antagonistic activity was estimated according to the measurement of the sterile halo around the agar wells in millimeters.

## 3. Results and Discussion

### 3.1. Material Characterization

**Figure 1:**
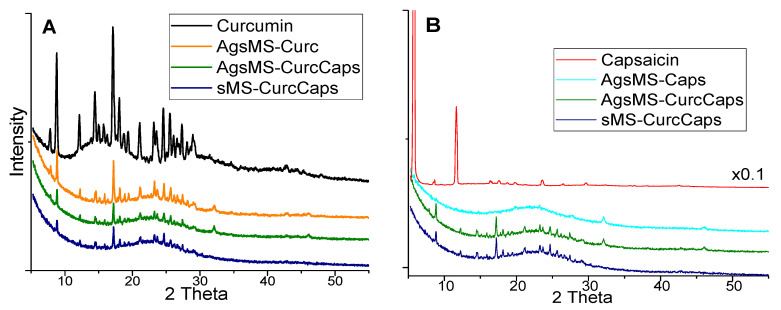
X-ray diffraction patterns of curcuminand curcumin-loaded pure and Ag-modified silica carriers (**A**), and capsaicinand capsaicin-loaded pure and Ag-modified silica carriers (**B**).

The high angle XRD diffractograms for Ag-containing spherical mesoporous silica and all obtained delivery systems are presented in Figure 1. For sMS and AgsMS samples, broad reflection is observed at 24° (Figure S1A), which confirms the amorphous state of the silica materials. The results indicated that the applied method for modification with Ag by exchange with the template leads to the preparation of materials containing silver monoxide (monoclinic structure according to card No. 43-1038, red markers in Figure S1B) as well as metallic silver nanoparticles (hexagonal structure according to card No. 41-1402, blue markers in Figure S1B). These reflections were not registered in the sMS diffractogram. The XRD data for pure curcumin (Figure 1A) showed sharp peaks at diffraction angles in the range of 8°–28° indicating its high crystalline nature. The patterns of all curcumin-loaded samples showed fewer intensive reflections for the crystalline form of the loaded compound due to its predominant loading into the pores in an amorphous state, and only a small amount of it can be found on the surface of the particles or in interparticle voids. The calculated Scherrer equation crystal sizes of the pure curcumin, curcumin loaded formulation, and bicomponent curcumin and capsaicin loaded formulation are 49, 68, and 62 nm, respectively. The formation of bigger crystals on the surface of the silica particles is probably a result of curcumin recrystallization during the procedure of loading and formation of dimers and network [61] of curcumin molecules, which can also block the entrances of the pores and hinder the pore feeling; however, the intensity of the reflections of the loaded curcumin is lower, assuming that only a small amount of it forms the crystalline phase. In Figure 1B), the diffractograms of pure capsaicin and its spherical mesoporous silica or AgsMS formulations are shown as well. Multiple sharp reflection signals of the pure drug are an indication of its crystalline nature, and the evaluated average crystal size is 68 nm; however, in the capsaicin-containing silica samples, no diffraction peaks (even the most intense around 6 and 12° 2*θ*) are present, which means that no capsaicin crystals are formed on the surface of the carriers. From the last observation, we can conclude that the whole amount of capsaicin is loaded into the pores of the carriers, or it is amorphized. The obtained delivery systems on the basis of Ag-modified carriers showed reflection at 32 and 45.2° 2*θ*, associated with the presence of AgO nanoparticles and Ag-nanoparticles or clusters, respectively, which are preserved during the BAC loading.

**Figure 2:**
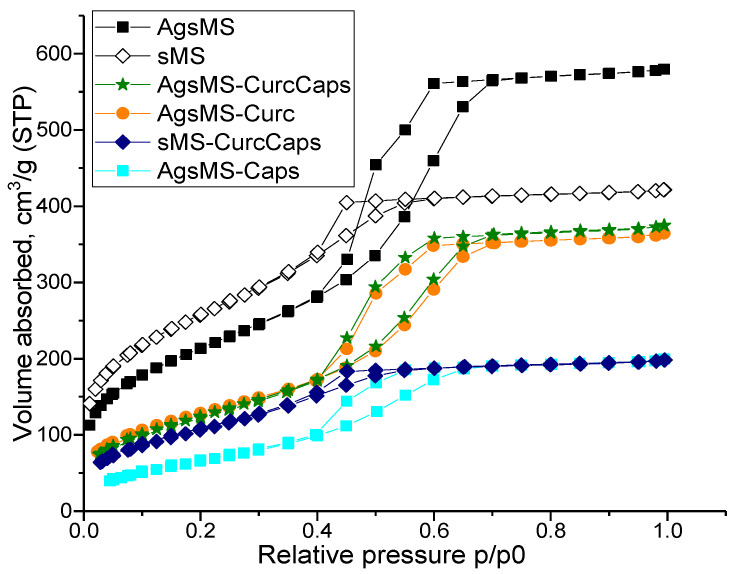
N_2_ physisorption data for the non-modified, Ag-modified silica supports and their curcumin and/or capsaicin-loaded analogs.

The results from the N_2_-physisorption measurements (Figure 2) showed type IV (IUPAC classification) isotherms for all of the studied porous carriers and obtained delivery systems and revealed their mesoporous structure. Presented isotherms are characterized bya hysteresis loop type H_2_, which is typical for the distribution of not well-defined pores with predominant bottleneck constrictions [54]. The calculated specific surface area, total pore volume, and pore size of all samples are summarized in Table 1. Both of the synthesized carriers (spherical mesoporous silica, Ag spherical mesoporous silica) exhibit significant loading capacity due to the high surface area and pore volume; however, it can be observed that the modification of parent spherical mesoporous silica with Ag bythe template ion-exchange procedure leads to the formation of bigger pores and higher total pore volume. A plausible explanation of this effect is the partial extraction of the template in more “soft” conditions during the ion-exchange procedure, in comparison with the high-temperature calcination, which leads to pore shrinkage in the case of parent silica material. The specific surface area of Ag-modified silica decreases by around 30%, compared with pure silica, not only because of the pore widening, but alsoprobably because of pore filling/blockage with silver nanoparticles. For the capsaicin-loaded carrier, a decrease ofaround 60% of the specific surface area is observed corresponding to pore filling, while for the curcumin, the decrease is only 30%, probably because of the formation of bigger polymeric-like molecule conjugates of curcumin molecules, which block the entrances of the pores leading to low pore feeling. These results are in good correspondence with the XRD observations.For the AgsMS sample containing both BACs decrease in the specific surface area is less pronounced in comparison with the samples loaded with one of the components. Our hypothesis is that the network of capsaicin and curcumin is formed between both types of molecules or in the presence of a solvent, and this limits their loading into the mesopores. Results supporting the described phenomenon are observed in the TG measurements as well.

**Figure 3:**
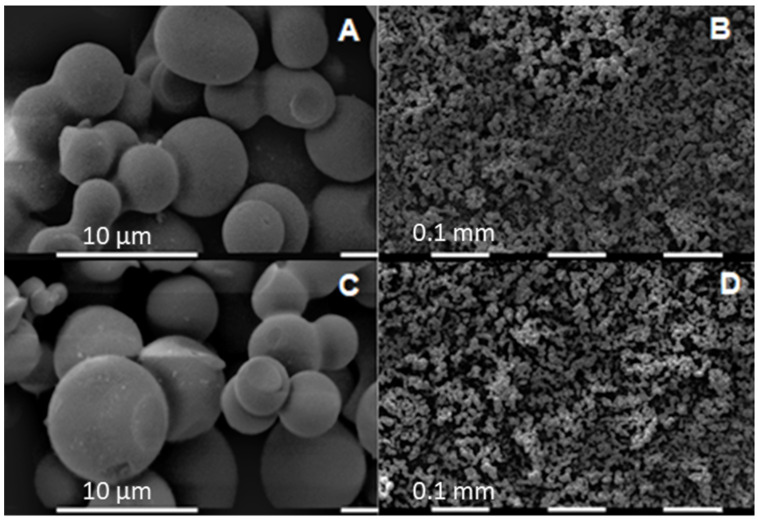
SEM images of non-modified (**A**,**B**) and Ag-containing (**C**,**D**) spherical mesoporous silica particles.

From the SEM micrographs presented in Figure 3A,B, it can be concluded that the used synthesis procedure leads to the formation of spherical silica particles with sizes between 5–8 µm (Figure S2A). In the images with higher magnetization, it can be noticed that some of the particles coalesced, probably during the gel formation, and solidified as bigger agglomerates; however, no changes in the particles’ morphology and size were noticed after the procedure of Ag-modification by impregnation (Figure 3C,D).

**Figure 4:**
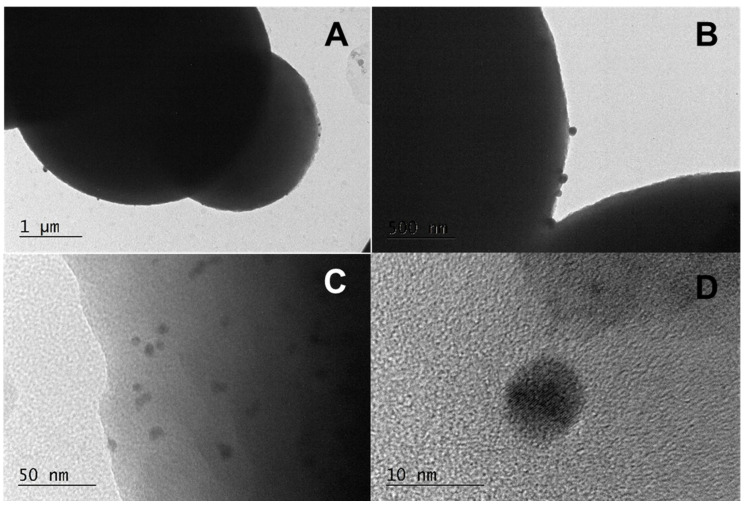
TEM images of the Ag-containing spherical porous carriers(**A**), fine dispersed small-sized silver species in the silica matrix (**C**), and bigger silver/silver oxide nanoparticles, with a size around 8 nm on the surface of the silica spheres (**B**,**D**).

TEM images of the Ag-containing porous carriers (Figure 4A) reveal the formation of spherical silica particles containing fine dispersed small-sized silver species in the silica matrix (Figure 4C and Figure S2B). From the TEM images can be seen that it is also possible to find bigger silver or silver oxide nanoparticleson the surface of the silica spheres or in between them (Figure 4B,D and Figure S2C).

**Figure 5:**
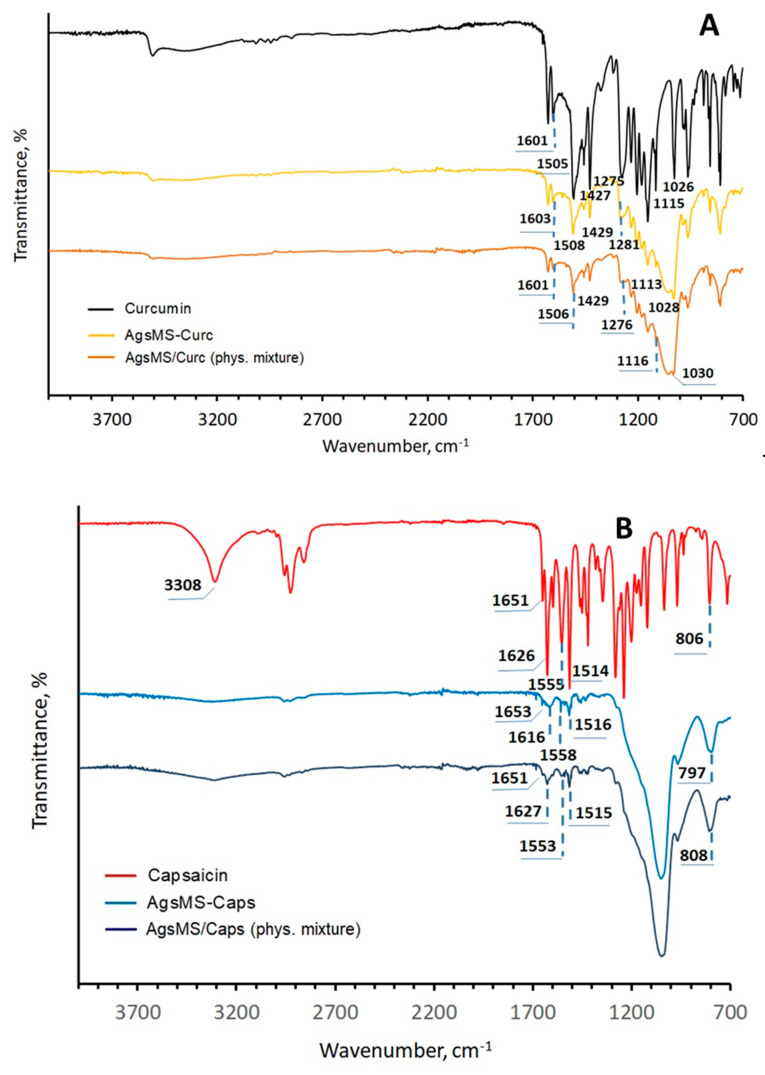
ATR-FTIR spectra of curcumin (**A**) or capsaicin (**B**) loaded modified sMS samples in comparison with free compounds and physical mixtures of support and BAC in the spectral region 4000 cm^−1^ to 700 cm^−1^.

In order to study in detail the interaction between the drugs’ molecules and their surroundings (the surface groups of the carriers), all curcumin and capsaicin impregnated parent silica and Ag-modified carriers were characterized by ATR-FTIR spectroscopy (Figure 5A,B). The formation of silica is confirmed by the strong signal between 1200 cm^−1^–1000 cm^−1^; nevertheless, this broadband does notcompromise the confirmation of the successful incorporation of curcumin and capsaicin into the mesoporous materials.

**Scheme 3:**
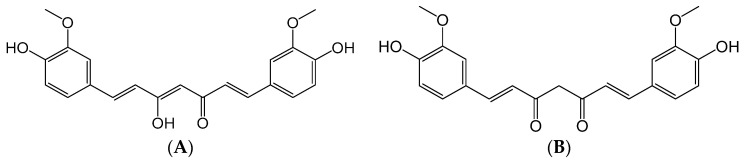
Chemical structure of curcumin molecule in enolic (**A**) and keto (**B**) form.

Curcumin ((1*E*,6*E*)-1,7-Bis(4-hydroxy-3-methoxyphenyl)hepta-1,6-diene-3,5-dione) is adiarylheptanoid, belonging to the group of natural phenols called curcuminoids. It is a keto–enoltautomer, existing inenolicform (Scheme 3A) in organic solvents and in keto form in water (quantitative approximation, because the curcumin is water-insoluble, Scheme 3B) [62]. Studies have shown that, in the crystalline phase, the enol configuration is preferred due to stabilization by a strong intramolecular H-bond formation [63].

The characteristic bands in the IR spectrum of pure curcumin are the plateau between 3540–3100 cm^−1^ due to the phenolic O–H stretching vibrations and formation of Hbonds, and the absorptions in the region from 3014 cm^−1^ to 2845 cm^−1^ assigned to the C–H stretchings, the unsaturated C=C and C=O groups of the inter-ring chain absorptions at 1626 cm^−1^ (ν(C=C) and ν(C=O)) while the band at 1601 cm^−1^ corresponds to the C=C stretching of the aromatic rings. The intensive band at 1505 cm^−1^ presents a combination of C=O stretching with CCC and CC=O bending vibrations. The absorption at 1427 cm^−1^ is due to CCC and CCH bendings and to a C–OH bending of the aromatic rings. In the region between 1275 cm^−1^ and 1024 cm^−1^, the aromatic C–O and C–O–C stretching vibrations are seen (Figure 5A) [64,65]. In the spectrum of the curcumin-loaded carrier (AgsMS-Curc), the bands of the bioactive compound are clearly observed through the intensive absorption due to the silica matrix. Moreover, the comparison of the spectrum of the pure curcumin and that of the loaded carrier displays indications for interaction between silica surface functionalities and curcumin molecules and curcumin molecules themselves. The spectral differences concern not only the region of the phenolic O–H stretching vibrations but also those associated with the double C=C and C=O bonds. Contribution to the spectral changes could produce the interactions of the silanol groups on the pore surface with the phenolic O–H groups and carbonyl oxygens of the bioactive compound. In addition, coordination of the latter with the Ag centers could be assumed [66]. The spectral changes include shifts of the absorptions at 1601 cm^−1^, 1505 cm^−1^, 1427 cm^−1^, 1275 cm^−1^, 1115 cm^−1^, and 1026 cm^−1^ to 1603 cm^−1^, 1508 cm^−1^, 1429 cm^−1^, 1281 cm^−1^, 1113 cm^−1^, and 1028 cm^−1^, respectively. Though the effects are not so pronounced due to the incorporation of the Ag nanoparticles in the silica matrix, they are consistent with the observations by Syed et al. [42]. The phenolic groups are responsible for the antioxidant activity of curcumin, which means that the interactions of these groups with the carrier can lead to decreased activity of the compound against free radicals.

**Scheme 4:**
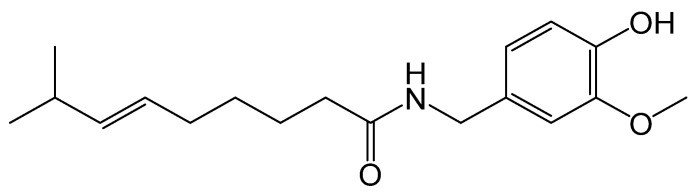
Chemical structure of capsaicin molecule.

Capsaicin ((6E)-*N*-[(4-Hydroxy-3-methoxyphenyl)methyl]-8-methylnon-6-enamide) is an alkaloid of the group of capsaicinoids, and its molecule contains three major fragments—aromatic head, amide linkage, and a hydrophobic tail (Scheme 4). The IR spectrum of the pure capsaicin is characterized by a broad band with a maximum at 3308 cm^−1^ due to N–H and O–H stretchings; absorptions in the region from 3016 cm^−1^ to 2800 cm^−1^ assigned to the C–H stretchings; the bands at: 1651 cm^−1^ (C=O stretching, Amide I), 1626 cm^−1^ (C=C stretching), 1555 cm^−1^ (aromatic C–C stretching), 1514 cm^−1^ (N–H bending and C–N stretching (Amide II)), 1422 cm^−1^ (CH_2_ bending), 806 cm^−1^ (out-of-plane C–H bending) and in the range 1283–1036 cm^−1^ (C–N, C–O, and C–O–C stretching vibrations), 970 cm^−1^ (C–C stretching) [67,68].

Similar to the case of curcumin, loading capsaicin in the Ag-modified carrier induces changes in the spectrum of the alkaloid. The XRD data revealed that the impregnation procedure led to the amorphization of capsaicin and its deposition into the pores of the silica carrier. This is supported by the FTIR spectral features of the AgsMS-Caps material. Because of the highly intensive absorption of the silica matrix, only the strongest bands of capsaicin are seen (Figure 5B). Nevertheless, spectral changes are observed in comparison to the spectrum of the pure alkaloid. The strong band due to N–H and O–H stretchings appears as a low-intensity broad plateau due to capsaicin interactions with the silanol groups and possibly with the Ag centers on the pore surface. The shift in C=O stretching (Amide I) can also be associated with interactions of this group with the silver species, similar to the published result obtained for capsaicin-coated silver nanoparticles [69]. Shifts of the other absorptions are also witnessed: Amide II bands were shifted by 2 cm^−1^, while the C=C stretching from 1626 cm^−1^ to 1616 cm^−1^, the aromatic ν(C–C) from 1555 cm^−1^ to 1558 cm^−1^, and the C–H bending from 806 cm^−1^ to 797 cm^−1^. The listed spectral changes prove the effectiveness of capsaicin loading and the interaction between the bioactive compound and the carrier surface.

FTIR spectra were performed onphysical mixtures of each bioactive substance with the carrier (Figure 5A,B). The characteristic bands of curcumin and capsaicin appeared at the same wavenumbers as in the spectra of the pure drugs, which is indirect proof that interaction between the silica surface and the molecules of both drugs occurred.

Formulations were prepared via simultaneous loading of curcumin and capsaicin from their mixed ethanolic solution via impregnation of the silica carrier. The FTIR spectra of the pure bioactive compounds and their physical mixture obtained via mixing of the solid compounds and after drying from their ethanolic solution are presented in Figure S3A,B. Differences in the spectral curve profiles in the region 3560–2800 cm^−1^ are seen between the spectra of the physical mixture and that obtained from the ethanol solution. The latter displays a broad plateau and increased intensity of the absorption at 2953 cm^−1^, which is attributed to residual ethanol bound to the two bioactive compounds via H-bonds. TGA analysis of the bicomponent formulation revealed that ethanol bridging the two bioactive molecules was present in the material. The FTIR spectra of the bicomponent formulation based on the spherical mesoporous silica and the Ag-modified carrier displayed similar spectral characteristics. The bands, due to the presence of curcumin, are more intensive;therefore, the absorptions of capsaicin are overlapped or not seen (Figure S3).

### 3.2. Loading Efficiency and In Vitro Release

**Figure 6:**
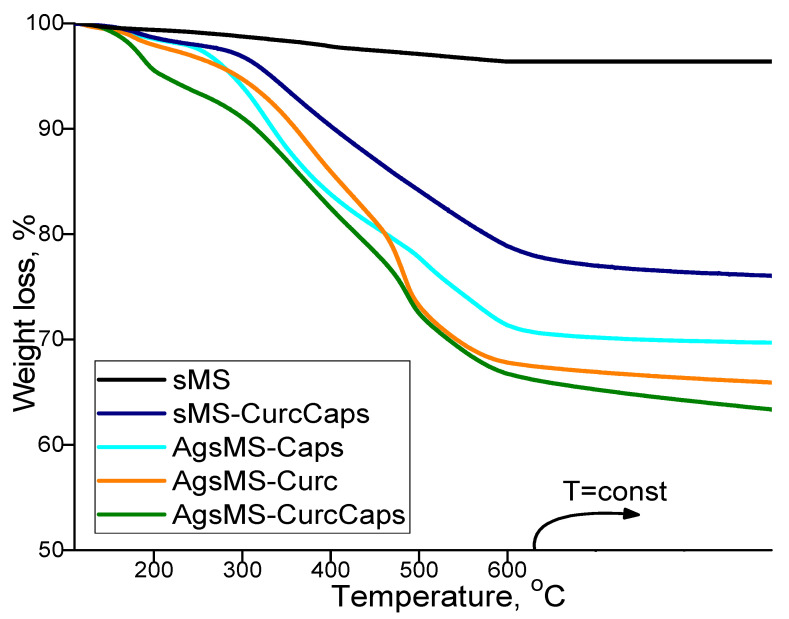
Thermogravimetric curves of obtained delivery systems treated in the air at a temperature from 110 to 600 °C.

The incipient wetness impregnation technique was applied to load BAC in pure or Ag-modified silica carriers. Ethanol was chosen as a solvent, due to its good solubilizing properties for curcumin and capsaicin, good toxicology profile, and easy evaporation at room temperature. BAC content in all elaborated drug delivery systems is determined by the thermogravimetric method, where the weight loss due to water removal for the parent silica sample was determinedup to 110 °C, and all the other samples were corrected according to this. Above this temperature, for the pure silica sample, less than 0.5% weight loss is observed, so the weight loss in all the other samples is assigned to the decomposition of the organic content (Curc and/or Caps). TG curves are presented in Figure 6 and the calculated data aresummarized in Table 1. The results showed effective loading of BAC in all the samples, with a percentage close to the theoretical calculated 33 wt.% based on the applied loading ratio BAC:carrier = 1:2. Only the sample based on non-modified spherical mesoporous silica showed dissimilarity from this trend and is characterized with only 22% loading, which can be explained by the narrow entrances of the pores and their easy blocking and hence hindered diffusion. It is interesting to mention that the simultaneously loaded with curcumin and capsaicin spherical non-modified and Ag-containing mesoporous silica formulations show significant weight loss (around 15 wt.%, not shown) up to 100 °C, most probably due to the loss of solvent, which could serve as a linker between BAC molecules. Such an effect is not observed for monocomponent formulations. We assume that the network of bonded capsaicin and curcumin molecules formed via solvent bridges restricts their penetration into the mesoporous channels, which could explain the low BACs loading into the silica carrier with narrow pores.

**Figure 7:**
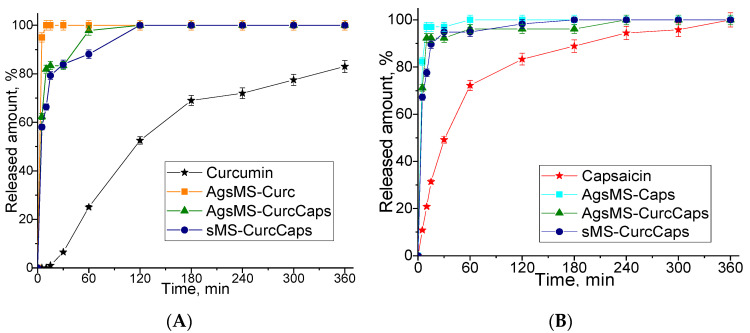
In vitro release profiles of curcumin (**A**) and capsaicin (**B**) loaded in non-modified and Ag-modified sMS in pH = 5.5.

The in vitrotests for curcumin and capsaicin release from the obtained delivery systems wasperformed in citratebuffer with pH = 5.5 (typical for dermal formulations) at 35 °C. The released amounts of Curc and Caps were shown in Figure 7A,B, respectively. For all samples on the basis of porous silica carriers, enhancement of the BAC solubility in buffer with pH = 5.5 was observed. During the monitored period (6 h), the effect was most pronounced (100% released amount in 15 min) for the system on the basis of Ag-modified silica. In the case of simultaneously loaded with both BACs silica nanocarriers, a bit slower release (almost 80% of the loaded drugs were released duringthe 120 min of the experiment) was observed, probably due to the formation of a network of both BACs molecules, which will slow down the diffusion in and out of the pores of the porous support.

### 3.3. Cytotoxicity

**Figure 8:**
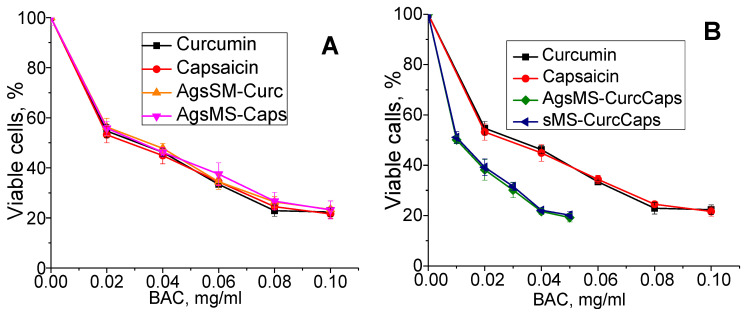
The growth inhibitory concentration–response curves as determined by the MTT-dye reduction assay after 72 h continuous exposure: (**A**) free vs. monocomponent loaded spherical mesoporous silica and (**B**) free BACs vs. simultaneously loaded curcumin and capsaicin. Each data point represents the arithmetic mean ± SD of 8 separate experiments.

The cell growth inhibitory effect of curcumin and/or capsaicin-loaded non-modified or Ag-modified sMS-based silica nanoparticles were evaluated in a comparative fashion vs. free BACs against HUT-78 (cutaneous T-cell lymphoma). A standard procedure, based on the enzymatic reduction of the yellow tetrazolium salt MTT to a violet MTT-formazan by the mitochondrial succinate dehydrogenase of vital cells, was used. The concentration-response curves are depicted in Figure 8, and the half inhibitory concentrations (IC_50_) derived thereof are shown in Table 2. As seen from the data, free drugs (as ethanol solutions) and their monocomponent spherical mesoporous silica formulations are characterized byconcentration-dependent cytotoxicity, although the antiproliferative effect of free drugs is slightly higher as compared to formulated ones. Thus, IC_50_ values of the non-modified or Ag-sMS loaded nanoparticles were slightly higher than those of the free drugs.

The lower reported activity of loaded BACs is probably due to the possible reabsorption of the initially released hydrophobic drugs onto the surface of the silica carriers, and therefore, the treated cells were exposed to lower concentrations of the drugs for the tested time period compared to the free curcumin and capsaicin.

Due to the similar molecular mechanism of the antitumor and anti-inflammatory effect of the two active substances curcumin and capsaicin, it was interesting to investigate their potential synergistic antitumor effect against HUT-78 cells. For this purpose, the cells were treated simultaneously and loaded with curcumin and capsaicin at 1:1 wt.% ratio unmodified or Ag-modified silicate nanoparticles. Thus, the cells were treated with a concentration of capsaicin and curcumin in the loaded nanoparticles (sMS-CurcCaps or AgsMS-CurcCaps) half lower than that of the solutions of free substances (1 mg/mL). The obtained results show that the simultaneously loaded sMS-CurcCaps and AgsMS-CurcCaps are characterized bythe higher antiproliferative activity of BAC even at 50% lower concentrations as compared to free drugs; thus, the combination of curcumin and capsaicin leads to potentiation of their antiproliferative effect as the reported IC_50_ values were 1.6 and 1.5 times lower than those of free curcumin and capsaicin, respectively. These results lead to the conclusion that the mixture of curcumin and capsaicin possesses a synergistic effect against studied cancerous cells. The observed pronounced antiproliferative activity of the elaborated curcumin- and capsaicin-loaded modified silica nanocarriers wereonly due to the antiproliferative activity of the two agents but not of the carriers themselves. The absence of cytotoxic potential of the empty carriers was demonstrated in our previous study against non-malignant cell line HEK-293 (human embryonic kidney cells), where even at the highest tested concentration, no suppression of cell viability was observed [70]. Considering the high loading efficiency and the potentiation of the antineoplastic activity of simultaneously loaded sMS nanoparticles, it can be concluded that they are promising systems for potential dermal application in various skin malignancies.

### 3.4. Antioxidant Activity

In order to determine whether the BAC retains itsantioxidant properties after itsincorporation into the mesoporous carriers, studies on the inhibition of 2,2-diphenyl-1-picrylhydrazyl (DPPH) free radical and ferric-reducing antioxidant power (FRAP) were performed (Table 3).

According to the radical scavenging activity assay, pure curcumin exhibited higher antiradical capacity than pure capsaicin. The mixture did notshow a synergistic effect against DPPH free radicals. The RSA against DPPH● of the mixture corresponds to the mean value of curcumin and capsaicin evaluated alone. Contrarily, pure capsaicin showed greater ferric-reducing antioxidant power than pure curcumin. A possible explanation for the lower ferric-reducing antioxidant power of pure curcumin is that in the case of the formation of dimers of this molecule, the reducing places are already involved in interactions, which hinder access to the big FRAP complex. The FRAP value obtained for the mixture of these biologically active substances revealed that they did not demonstrate synergism in respect of their ferric-reducing antioxidant power. The mixture’s reducing power was even lower than the average of the pure compound’s (and closer to this of the weak FRAP active compound–curcumin).

The results revealed that the ferric-reducing antioxidant power of AgsMS-Caps decreased by only 3.77% compared to pure capsaicin. The radical scavenging activity of this sample against DPPH didnot change significantly, so it could be assumed that the impregnation of the capsaicin into the AgsMS carrier by the incipient wetness impregnation method did not affect the properties of the active compound.

The FRAP value of sMS-CurcCaps shows a negligible decrease (only 5.16%) compared with the mixture 1:1 Curc:Caps, and the antiradical activity of the curcumin–capsaicin mixture increased only from 24.27 ± 0.27% to 26.51 ± 0.23% after its loading into the non-modified silica carrier. These results confirmed that the loading of the mixture of curcumin and capsaicin in a non-modified silica carrier preserves their radical scavenging and antioxidant activity.

The most pronounceddecrease in the antioxidant properties of the active compounds was observed for the BACs-loaded AgsMS. The radical scavenging activity of curcumin and the curcumin–capsaicin mixture in the systems AgsMS-Curc and AgsMS-CurcCaps falls down from 31.61 ± 0.25% to 27.37 ± 0.29% and from 24.27 ± 0.27% to 17.13 ± 0.27%, and the FRAP values decreased with 14.56% and 38.14%, respectively. The observed decrease in the antioxidant properties of the systems on the basis of Ag-modified sMS, containing only curcumin and the curcumin–capsaicin mixture, could be a result of an Ag-curcumin complex and curcumin dimers formation, which is in accordance with the results from IR analysis and result for such complexes reported previously in the literature [42]. A more significantly pronounced decrease in the antioxidant properties of Ag-containing curcumin systems, in comparison with the ones with capsaicin, could be explained by a blocking of the moieties in the BAC molecule that are responsible for this activity. The stronger effect in the system containing both BACs could be a result of additional interaction between the molecules of curcumin and capsaicin.

### 3.5. In Vitro Antibacterial Activity

The antibacterial activity of the pure bioactive components, AgsMS and its curcumin and capsaicin-loaded varieties, was tested against threepathogenic microorganisms: *Escherichia coli* ATCC 25922, *Pseudomonas aeruginosa* ATCC 9027, and *Staphylococcus aureus* ATCC 6538; the results are presented in Table 4. The pure bioactive components (curcumin and capsaicin) demonstrated very low antibacterial activity. The Ag-containing mesoporous silica displayed certain activity, as evidenced by the broadest sterile halo among the tested samples. The effect was not so strong due to the incorporation of the Ag nanoparticles in the silica matrix. Nevertheless, the presence of Ag in the carrier enhanced the antibacterial activity of the curcumin and capsaicin-loaded AgsMS formulations. The latter exhibited a two to three times increase inthe inhibition zone in comparison to the pure bioactive components.

## 4. Conclusions

Spherical mesoporous silica carriers with high specific surface area (904 m^2^/g) and pore volume (above 0.6 cc/g) were synthesized and modified with silver by the templateion-exchange method. Using theincipient wetness technique, the obtained materials were impregnated with curcumin, capsaicin, or a mixture of both (up to 33%). The developed delivery systems, as well as the pure silica carriers, were fully characterized to clarify the role of the modification procedure on the textural and physicochemical properties of the materials. It was found that the exchange with the template as a procedure for modification and removal of the organic structure-directing agent, leads to materials with wider pores, in comparison with the most commonly used calcination method. ATR-FTIR spectroscopic data suggested weak interaction of curcumin and capsaicin with the surface of non- and Ag-modified supports. The results from in vitro release tests for curcumin and capsaicin revealed that the loading in porous silica carriers enhances their solubility in buffer with pH 5.5. For the delivery systems of capsaicin and curcumin on the basis of non-modified silica, the radical scavenging activity against DPPH stable free radicals and the ferric-reducing antioxidant power of the loaded biologically active compounds are not compromised; however, for the delivery systems on the basis of Ag-modified sMS, containing curcumin and curcumin–capsaicin mixture, the RSA and the FRAP values decrease, which could be an effect of an Ag–curcumin or capsaicin–Ag–curcumin complexes formation. A comparative study of the cytotoxic potential of free and formulated biologically active compounds and the mixture of them showed that the system for simultaneous delivery of both drugs has at least 1.5 times lower IC_50_ values than the free curcumin and capsaicin. Results from antibacterial activity tests showed that AgsMS formulations have two to three times higher inhibition zone in comparison to the pure bioactive components. Considering the excellent results from the antineoplastic activity, antioxidant activity, and antibacterial activity tests of the obtained delivery systems on the basis of the silica nanoparticles loaded with curcumin and capsaicin, we can conclude that these systems are promising for potential dermal application in the treatment of skin conditions and disorders.

## Figures and Tables

**Table 1:** Textural characteristics of the initial mesoporous silicas and the BAC loaded formulations and the content of BAC.

Sample	Specific Surface Area, m^2^/g	Total Pore Volume, cc/g	Pore Size nm	Content of BAC, wt.%
sMS	904 ± 11	0.65 ± 0.008	3.1 ± 0.04	-
AgsMS	625 ± 8	0.75 ± 0.009	5 ± 0.05	-
AgsMS-Caps	258 ± 3	0.31 ± 0.004	4.3 ± 0.05	29
AgsMS-Curc	447 ± 5	0.57 ± 0.006	4.9 ± 0.06	32
AgsMS-CurcCaps	460 ± 6	0.58 ± 0.006	4.9 ± 0.06	33
sMS-CurcCaps	397 ± 5	0.31 ± 0.004	3.1 ± 0.04	22

**Table 2:** Equieffective concentrations (IC_50_) of free and loaded BACs.

	Cell Line	HUT-78 IC_50_, mg/mL
Sample		
Curcumin		0.030
Capsaicin		0.028
AgsMS-Curc		0.035
AgsMS-Caps		0.032
AgsMS-CurcCaps		0.019
sMS-CurcCaps		0.021

**Table 3:** RSA and FRAP of the tested parent and modified carriers loaded with pure curcumin, capsaicin, and curcumin–capsaicin mixture used as positive controls.

Samples	DPPH RSA, %	FRAP Value, µmol Fe^2+^/L
Curcumin	31.61 ± 0.25	736.7 ± 0.0
Capsaicin	19.46 ± 0.84	981.1 ± 28.4
Curcumin:Capsaicin 1:1 (*w*/*w*)	24.27 ± 0.27	790.6 ± 5.9
AgsMS-Curc	27.37 ± 0.29	629.4 ± 6.6
AgsMS-Caps	18.69 ± 0.63	944.1 ± 1.9
AgsMS-CurcCaps	17.13 ± 0.27	489.1 ± 2.3
sMS-CurcCaps	26.51 ± 0.23	749.8 ± 13.1

Each assay was carried out in triplicate. The experimental data are presented as mean ± standard deviation. The concentration of the pure compounds loaded in the carriers corresponds to 100 μg/mL.

**Table 4:** Antibacterial activity of new nanomaterials against Gram-negative and Gram-positive microorganisms at the 24th hour. Experiments were performed in triplicate and the average value was determined.

Samples	Concentration (mg/mL)	Antibacterial Activity (mm of the Sterile Halo)		
		*E. coli*	*P. aeruginosa*	*S. aureus*
Capsaicin	5 *	1	0.5	1.5
Curcumin	5 *	1	1	1
AgsMS-Curc	20 **	2	1.5	2
AgsMS-Caps	19 **	2.8	2.5	2.9
AgsMS	15	4.5	4.5	4

* The concentrations are comparable to those in the loaded formulations. ** These concentrations correspond to a concentration of 15 mg/mL for the Ag-containing mesoporous silica.

## Data Availability

Not applicable.

## Supplementary Materials

The following supporting information can be downloaded at: https://www.mdpi.com/article/10.3390/nano12173075/s1, Figure S1. XRD patterns of parent sMS and AgsMS samples (A) and XRD patterns of Ag-containing silica and corresponding Ag and AgO reflections (B), Figure S2. Histogram of Si-particles size distribution from images SEM (A) and histograms for Ag-particles size distribution from TEM images (B,C)., Figure S3. ATR-FTIR spectrum of pure curcumin and capsaicin and not loaded mixture (A) and spectrum of curcumin and capsaicin mixtures not loaded and loaded in parent and Ag-modified silica (B).
