# Supplementary material for: Curcumin and Capsaicin-Loaded Ag-Modified Mesoporous Silica Carriers: A New Alternative in Skin Treatment
Source: Nanomaterials (Basel). 2022 Sep 5;12(17):3075. doi: 10.3390/nano12173075 (PMC9458240; doi:10.3390/nano12173075)
Supplement: Supplementary file 1 [file nanomaterials-12-03075-s001.zip › nanomaterials-1857836-supplementary.pdf]

Curcumin and Capsaicin-Loaded Ag-Modified Mesoporous Silica Carriers: A New Alternative in Skin Treatment

Ivalina Trendafilova[1,*], Ralitsa Chimshirova[1], Denitsa Momekova[2], Hristo Petkov[1], Neli Koseva[3], Penka Petrova[4] and Margarita Popova[1]

[1] Institute of Organic Chemistry with Centre of Phytochemistry, Bulgarian Academy of Sciences, 1113 Sofia, Bulgaria

[2] Faculty of Pharmacy, Medical University of Sofia, Sofia, 1000, Bulgaria

[3] Institute of Polymers, Bulgarian Academy of Sciences, 1113 Sofia, Bulgaria

[4] Institute of Microbiology, Bulgarian Academy of Sciences, Sofia, 1113, Bulgaria;

* Correspondence: Ivalina.Trendafilova@orgchm.bas.bg

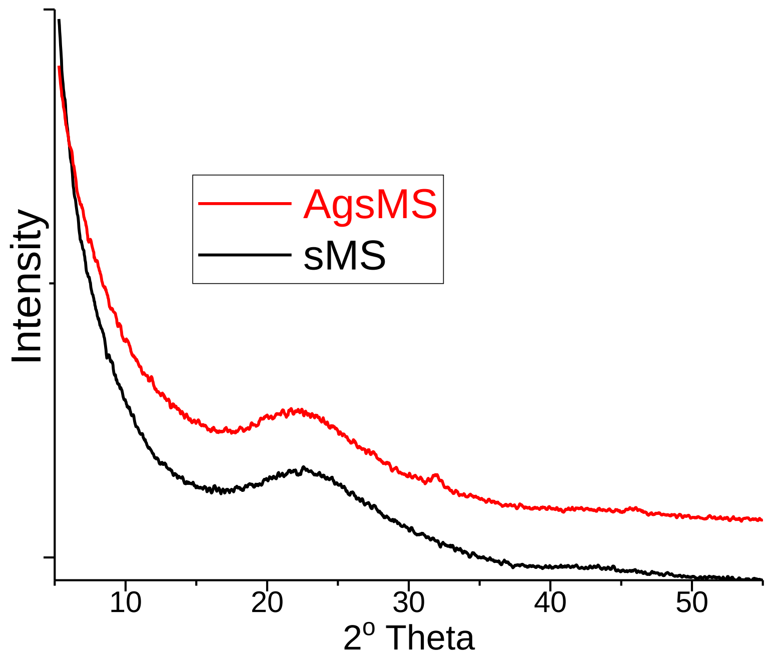

(A)

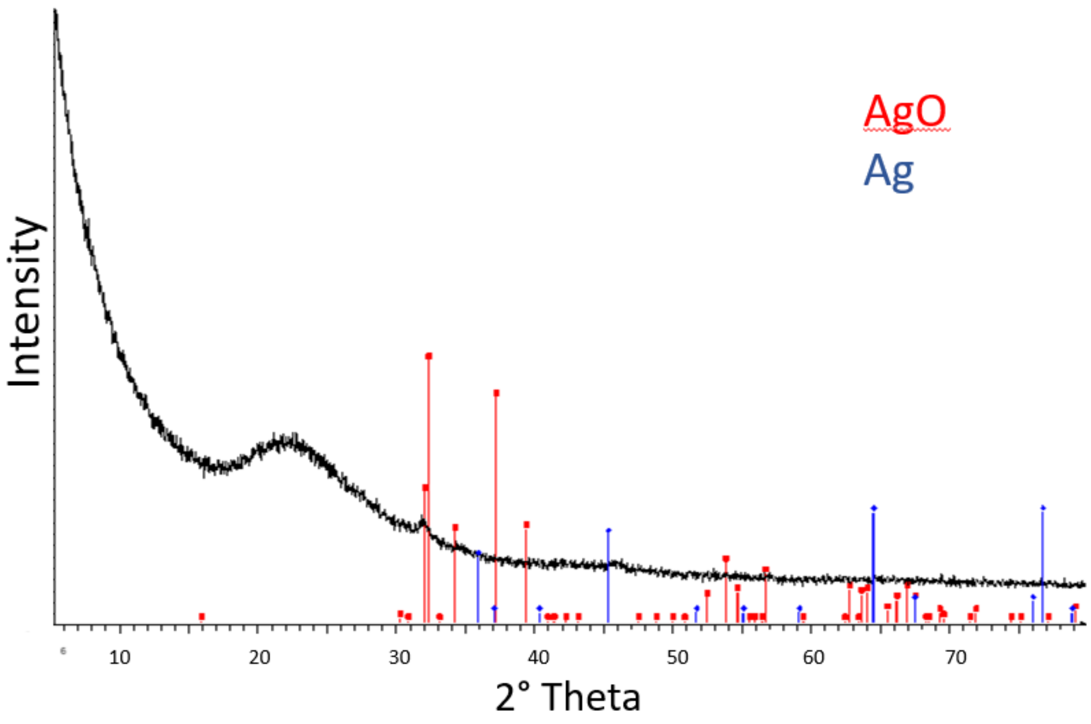

(B)

Figure S1. XRD patterns of parent sMS and AgsMS samples (**A**) and XRD patterns of Ag-containing silica and corresponding Ag and AgO reflections (**B**)

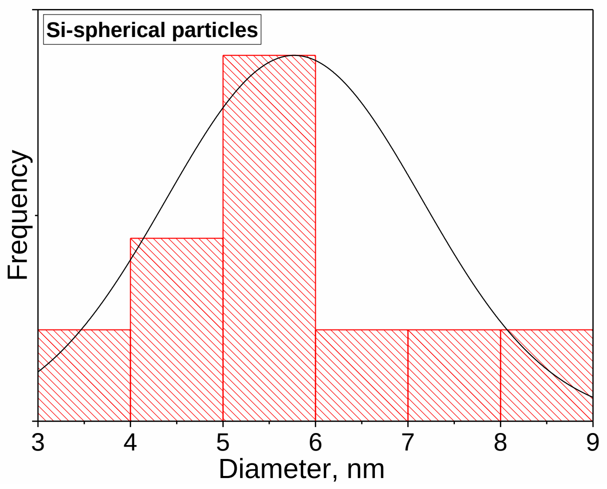

(A)

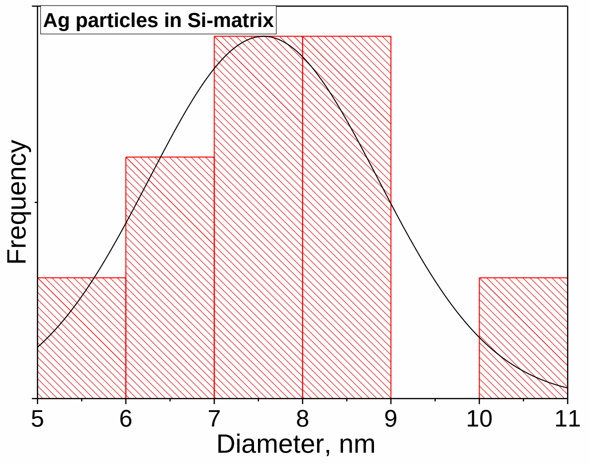

(B)

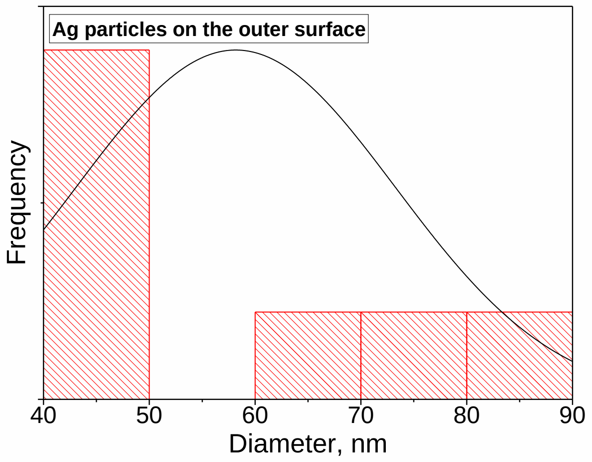

(C)

Figure S2. Histogram of Si-particles size distribution from images SEM (**A**) and histograms for Ag-particles size distribution from TEM images (**B**,**C**)

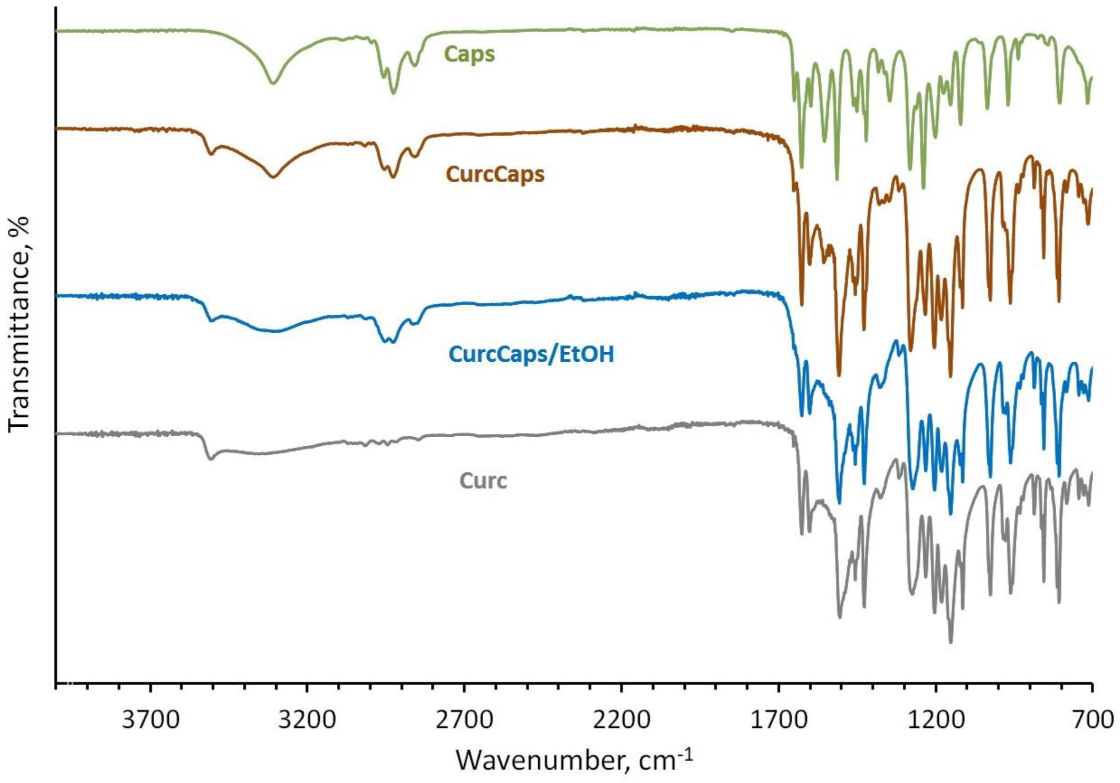

(A)

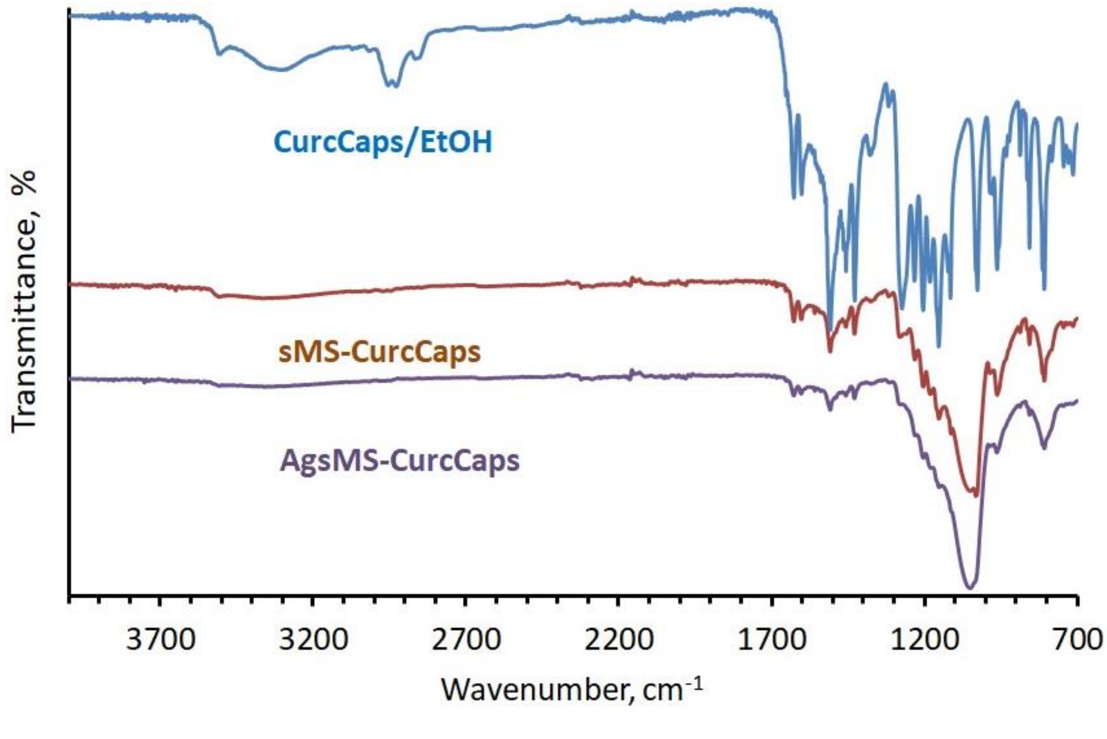

(B)

Figure S3. ATR-FTIR spectrum of pure curcumin and capsaicin and not loaded mixture (**A**) and spectrum of curcumin and capsaicin mixtures not loaded and loaded in parent and Ag-modified silica (**B**)
